# *In vitro* study to assess shear bond strength of e-mineral trioxide aggregate with glass ionomer cement

**DOI:** 10.6026/973206300213301

**Published:** 2025-09-30

**Authors:** Parthivi Singh, Anupama Ahirwar, Ananya Bhargava, Apoorva Sharma, Subasish Behera, Soumyaranjan Nanda, Miral Mehta, Dhirendra Kumar Singh

**Affiliations:** 1 Department of Conservative Dentistry and Endodontics, Peoples Dental Academy, Bhopal, Madhya Pradesh, India; 2 Department of Dentistry, Sunderlal Patwa Government Medical College, Mandsaur, Mathya Pradesh India; 3 Consultant Orthodontist, Avanti Hospital, Ujjain, Madhya Pradesh, India; 4 Department of Conservative Dentistry and Endodontics, Triveni Institute of Dental Sciences, Hospital & Research Centre, Bodri, Chhattisgarh, India; 5 Department of Conservative Dentistry and Endodontics, Government Dental College and Hospital (SCB), Manglabag, Cuttack, Odisha, India; 6 Endodontics, Private Practitioner, Cuttack, Odisha, India; 7 Department of Pediatric and Preventive Dentistry, Karnavati School of Dentistry, Karnavati University, Gandhinagar, Gujarat, India; 8 Department of Periodontology, Kalinga Institute of Dental Sciences, KIIT Deemed to be University, Bhubaneswar, India

**Keywords:** Mineral trioxide aggregate, glass ionomer cement, shear bond strength, endodontic restoration, adhesion timing

## Abstract

The shear bond strength (SBS) between enhanced Mineral Trioxide Aggregate (e-MTA) and glass ionomer cement (GIC) under different placement intervals is of interest. Sixty acrylic blocks filled with e-MTA were restored with GIC at 0 min, 15 min and 24 h. The highest SBS (12.47 ± 1.29 MPa) was observed at the 15-minute interval. Predominantly mixed failures were seen in Groups II and III, while cohesive failures occurred in Group I. Applying GIC 15 minutes after e-MTA yields optimal bonding for durable restorations.

## Background:

Mineral trioxide aggregate (MTA) is hydrophilic, biocompatible endodontic cement predominantly composed of tricalcium silicate, dicalcium silicate, tricalcium aluminate and bismuth oxide as a radiopacifier. It exhibits excellent sealing ability, dimensional stability and the capacity to set in the presence of moisture, making it suitable for various clinical applications in endodontics and restorative dentistry [1]. Originally introduced for retrograde root-end fillings, MTA has shown remarkable versatility and has since been employed in a wide array of procedures including direct and indirect pulp capping, apexification, repair of root and furcation perforations, internal resorption and regenerative endodontics due to its high pH (~12.5), bioactive potential and stimulation of hydroxyapatite formation [2]. On the other hand, conventional glass ionomer cement (GIC) has long served as a fundamental restorative material owing to its ability to chemically bond to dentin and enamel, its sustained fluoride release, thermal compatibility with tooth structure and ease of handling. GIC's acid-base setting reaction and the formation of an ion-exchange layer enhance the marginal seal and reduce microleakage, contributing to its continued clinical relevance [3]. In scenarios such as vital pulp therapy or perforation repair, layering GIC over MTA is frequently employed to create a hermetic coronal seal and provide mechanical support.

While studies have investigated the interfacial bonding characteristics of MTA with composite resins and resin-modified glass ionomer cements (RMGICs), outcomes have often varied based on surface treatments, timing of placement and MTA type-conventional or fast-set variants [4, 5]. The shear bond strength (SBS) of these combinations is pivotal in ensuring the longevity of restorations and preventing marginal failure or debonding under functional stresses. However, literature focused specifically on the bond strength between newer enhanced MTA (e-MTA) formulations and conventional GIC remains limited and the role of timing in achieving optimal adhesion has not been fully elucidated. Given the setting dynamics of MTA, especially during its initial hydration phase, premature application of GIC may disrupt the crystalline matrix or result in interfacial weakness. Conversely, delayed placement might reduce chemical interlocking potential due to the formation of a mature surface. Determining the ideal window for GIC placement over e-MTA is therefore critical for optimizing adhesion and ensuring clinical predictability [6]. Therefore, it is of interest to evaluate the shear bond strength between e-MTA and conventional GIC when GIC is applied immediately, 15 minutes after and 24 hours after e-MTA placement under standardized *in vitro* conditions. This investigation seeks to inform evidence-based protocols for restorative layering over MTA, thereby improving clinical outcomes in endodontic and restorative procedures.

## Materials and Methods:

This experimental, randomized *in vitro* study was designed to evaluate the shear bond strength (SBS) between enhanced mineral trioxide aggregate (e-MTA) and conventional glass ionomer cement (GIC) under controlled laboratory conditions. A total of sixty standardized acrylic resin blocks were fabricated, each measuring 20 mm in length, 20 mm in width and 5 mm in height. A central cylindrical cavity of 4 mm in diameter and 2 mm in depth was prepared in each block using a precision milling device. These blocks were randomly assigned into three equal groups (n=20) based on the timing of GIC placement over e-MTA: Group I (immediate application), Group II (15 minutes post-placement) and Group III (24 hours post-placement). The materials used included enhanced mineral trioxide aggregate (Kids-E-Dental, India), conventional GIC (Fuji IX GP, GC Corporation, Japan), distilled water, a humidity chamber and a universal testing machine (Instron 3345, Instron Corp., USA). For the experimental procedure, e-MTA was prepared by mixing powder and liquid at a standardized powder-to-liquid ratio of 3:1 on a glass slab, as per the manufacturer's guidelines. The freshly mixed e-MTA was then placed into the prepared cavities in the acrylic blocks and gently condensed with a non-absorbent plugger to ensure complete filling and surface levelling. Following placement, all specimens were stored in a humid environment maintained at 100% relative humidity and 37°C to simulate intraoral conditions. In Group I, the GIC was applied immediately after e-MTA placement. In Group II, the GIC application was performed 15 minutes after e-MTA placement, while in Group III, the GIC was applied after 24 hours. Conventional GIC was prepared according to manufacturer instructions and loaded into custom-made cylindrical plastic tubes (4 mm diameter x 2 mm height), which were positioned vertically onto the set or semi-set e-MTA surface. The GIC was allowed to set for 10 minutes at 37 °C in the humidity chamber before removing the tubes. Shear bond strength testing was performed using the Instron 3345 universal testing machine. Each specimen was mounted securely and a knife-edged loading blade was positioned precisely at the e-MTA-GIC interface. A shear load was applied at a crosshead speed of 0.5 mm/min until bond failure occurred. The peak load at failure was recorded in Newtons (N) and subsequently converted to megapascals (MPa) by dividing the load by the bonding area. Following mechanical testing, the debonded surfaces were examined under a stereomicroscope at 20x magnification to determine the mode of failure. Failures were categorized as adhesive (failure at the e-MTA/GIC interface), cohesive (failure within e-MTA or GIC), or mixed (combination of adhesive and cohesive). For statistical analysis, one-way analysis of variance (ANOVA) was employed to compare mean SBS values across the three groups. Post hoc comparisons were performed using Tukey's multiple comparison test. A significance level of α = 0.05 was set for all statistical tests. Additionally, chi-square analysis was conducted to evaluate the distribution of failure modes among the groups. All statistical procedures were executed using standard statistical software (e.g., SPSS version 25.0).

## Results:

The timing of GIC placement over e-MTA significantly influence shear bond strength values. Immediate placement (Group I) showed the lowest bond strength (7.82 ± 1.15 MPa), which was statistically inferior to both 15 min and 24 h groups. After a 15-minute interval (Group II), the bond strength was highest (12.47 ± 1.29 MPa), significantly greater than both immediate and 24 h placement. At 24 h (Group III), bond strength (10.03 ± 1.02 MPa) was higher than immediate placement but lower than the 15-minute group. Mean shear bond strength values (MPa ± SD) are presented in Table 1 (see PDF). In Table 2 (see PDF), failure mode analysis revealed distinct patterns across groups. In group I, failures were predominantly cohesive (65%), with fewer adhesive (30%) and mixed failures (5%). Group II showed a markedly different distribution, with mixed failures dominating (70%), while adhesive and cohesive failures were equally low (15% each). In Group III, mixed failures were also common (55%), followed by adhesive (25%) and cohesive failures (20%). Group II exhibited predominantly mixed failures, indicating robust interfacial integrity.

## Discussion:

The present study demonstrates that the application of conventional glass ionomer cement (GIC) 15 minutes after placement of enhanced mineral trioxide aggregate (e-MTA) yields the highest shear bond strength (SBS), averaging 12.47 MPa. Although this value approaches the lower limit of the clinically acceptable threshold for gap-free restoration margins (17-20 MPa), it does not fully satisfy it, indicating partial fulfilment of ideal clinical bonding requirements [7]. The significantly lower SBS observed in Group I (immediate application) can be attributed to the insufficient primary setting reaction of e-MTA, which compromises interfacial integrity. During the initial hydration phase, the material remains susceptible to moisture displacement and surface disintegration, which likely undermines effective adhesion with GIC [8]. Interestingly, delaying GIC application by 24 hours (Group III) improved bond strength over immediate placement but did not surpass the 15-minute interval. This supports the hypothesis that a semi-set state of e-MTA, occurring around 15 minutes post-mix, provides optimal conditions for micromechanical interlocking and potential ionic exchange between the calcium-rich surface of MTA and the polyalkenoic acid matrix in GIC [9]. In this transitional phase, the partially matured surface retains sufficient porosity and moisture for interfacial reactions without compromising its structural stability. The results match the previous studies on resin-modified GIC (RMGIC) and ProRoot MTA, in which the bond strengths gradually increased over a number of days because of the continued hydration process of MTA and the precipitation of hydroxyapatite-like lattices at the interfaces. These precipitates promote a bioactive layer favorable in chemical bonding with polyacid-based materials such as GIC [5]. The failure mode analysis also confirms the mechanical trends: Group I mostly demonstrated cohesive failures in the e-MTA itself, indicating that the substrate was not considered to have integrity at the early stages. By contrast, Groups II and III exhibited mixed failure modes, which suggested a more positive effective substrate cohesive strength and adhesive perspective strength of interfacial bonding. These results support other works that highlight the role of the timing of application on layered restorative procedures with calcium silicate-based cements and glass ionomer-based products [10, 11]. The critical time seems to be 15 minutes, as it will ensure the maximum efficacy of the adhesive and will not expose the substrate to dissolve and degrade it or reduce the chemical connection [12, 13]. This investigation has such strengths as a standardized *in vitro* model, consistent specimen geometry and comprehensive failure pattern analysis, which has increased internal validity. However, the research has its own a few limitations. They have not used thermocycling and long-term aging, as well as cyclical loading, which are common tests in bond longevity and clinical performance. Such factors are critical in the strength of restorative bonds in the dynamic oral environment [14, 15-16]. These limitations should be overcome in future studies by introducing thermal fatigue protocols and mechanical aging cycles. Moreover, trial of surface modification methods like mild acid etching, sandblasting, or utilization of intermediate bonding agents would have also resulted in additional improvement of SBS and overall long-term interface stability. The effect of various GIC viscosities and other calcium silicate formulations should also be explored to increase the clinical applicability of such findings [17].

## Conclusion:

Applying GIC 15 minutes after e-MTA placement optimizes shear bond strength, facilitating a durable interface for restorative procedures. Hence, clinicians should consider this time window to ensure reliable adhesion when combining e-MTA with conventional GIC.
